# Supplementary material for: Iterative improvement in the automatic modular design of robot swarms
Source: PeerJ Comput Sci. 2020 Dec 7;6:e322. doi: 10.7717/peerj-cs.322 (PMC7924708; doi:10.7717/peerj-cs.322)
Supplement: Supplemental Information 3 [file peerj-cs-06-322-s003.zip › argos3/doc/api/standalone/a00419_source.html]

ARGoS: plugins/robots/e-puck/simulator/qtopengl\_epuck.h Source File


- Main Page
- Related Pages
- Namespaces
- Classes
- Files

- File List
- File Members

# plugins/robots/e-puck/simulator/qtopengl\_epuck.h

Go to the documentation of this file.

```
00001 
00007 #ifndef QTOPENGL_EPUCK_H
00008 #define QTOPENGL_EPUCK_H
00009 
00010 namespace argos {
00011    class CQTOpenGLEPuck;
00012    class CEPuckEntity;
00013 }
00014 
00015 #ifdef __APPLE__
00016 #include <gl.h>
00017 #else
00018 #include <GL/gl.h>
00019 #endif
00020 
00021 namespace argos {
00022 
00023    class CQTOpenGLEPuck {
00024 
00025    public:
00026 
00027       CQTOpenGLEPuck();
00028 
00029       virtual ~CQTOpenGLEPuck();
00030 
00031       virtual void Draw(CEPuckEntity& c_entity);
00032 
00033    protected:
00034 
00036       void SetGreenPlasticMaterial();
00038       void SetRedPlasticMaterial();
00040       void SetCircuitBoardMaterial();
00042       void SetLEDMaterial(GLfloat f_red,
00043                           GLfloat f_green,
00044                           GLfloat f_blue);
00045 
00047       void RenderWheel();
00049       void RenderChassis();
00051       void RenderBody();
00053       void RenderLED();
00054 
00055    private:
00056 
00058       GLuint m_unLists;
00059 
00061       GLuint m_unWheelList;
00062 
00064       GLuint m_unChassisList;
00065 
00067       GLuint m_unBodyList;
00068 
00070       GLuint m_unLEDList;
00071 
00074       GLuint m_unVertices;
00075 
00076       /* Angle gap between two leds */
00077       GLfloat m_fLEDAngleSlice;
00078 
00079    };
00080 
00081 }
00082 
00083 #endif
```

---

Generated on 10 Jul 2018 for ARGoS by 
 1.6.1
